# Trends in brachytherapy utilization in Canada from 2011 to 2020

**DOI:** 10.1016/j.phro.2026.100974

**Published:** 2026-04-30

**Authors:** Mirta Dumančić, Yujing Zou, Harry Glickman, Sara Ghassimi Kouraneh, Piotr Pater, Andrew Alexander, Jorge E. Alpuche Aviles, Amanda Cherpak, Kristin Marchant, Geetha Menon, Daniel Morton, Tim Olding, Moti Paudel, Alexandra Rink, Ingrid Spadinger, Shirin A. Enger

**Affiliations:** a Medical Physics Unit, Department of Oncology, Faculty of Medicine, McGill University, Montréal, QC, Canada; b Lady Davis Institute for Medical Research, Montréal, QC, Canada; c Faculty of Health Sciences, Simon Fraser University, Burnaby, BC, Canada; d Department of Radiation Oncology, Queen Elizabeth Hospital, Prince Edward Island Cancer Treatment Centre, Charlottetown, PEI, Canada; e Department of Physics & Engineering Physics, University of Saskatchewan, Saskatoon, SK, Canada; f Department of Radiology and Physics & Astronomy, University of Manitoba, Winnipeg, MB, Canada; g Medical Physics, Cancer Care Manitoba, Winnipeg, MB, Canada; h Department of Medical Physics, QEII Health Sciences Centre, Halifax, NS, Canada; i Allan Blair Cancer Centre, Department of Medical Physics, Regina, SK, Canada; j Department of Oncology, Cross Cancer Institute, University of Alberta, Edmonton, AB, Canada; k Saskatoon Cancer Centre, Department of Medical Physics, Saskatoon, SK, Canada; l Medical Physics, Kingston Health Sciences Centre, Kingston, ON, Canada; m Department of Medical Physics, Sunnybrook Health Sciences Centre, Toronto, ON, Canada; n Department of Radiation Oncology, University of Toronto, Toronto, ON, Canada; o Department of Medical Biophysics, University of Toronto, Toronto, ON, Canada; p Princess Margaret Cancer centre, University Health Network, Toronto, ON, Canada; q BC Cancer (Vancouver), Department of Medical Physics, Vancouver, BC, Canada; r Research Institute of the McGill University Health Centre, Montréal, QC, Canada

**Keywords:** Brachytherapy utilization, High-dose-rate, Low-dose-rate, Canada, Radiation oncology, Healthcare policy

## Abstract

**Background and Purpose:**

Brachytherapy is a highly conformal and cost-effective radiotherapy modality, yet its clinical utilization has declined in multiple regions. This study investigated trends in brachytherapy utilization across different Canadian provinces from 2011 to 2020.

**Materials and Methods:**

A national survey was distributed to medical physicists in cancer centres across ten Canadian provinces, collecting data on types of brachytherapy, annual number of treatments, clinical indications, and logistical factors. In Québec, treated clinical indications were obtained through a complementary survey of all radiotherapy centres, extending previously published provincial brachytherapy data.

**Results:**

Out of 39 radiotherapy centres in Canada conducting brachytherapy treatments, 25 centres were included in this study. HDR accounted for the majority of treatments (60%–100%) and increased in most provinces, while LDR declined across reporting provinces; PDR comprised ≤10% of treatments and was limited to Alberta and Ontario. After adjusting for indication-specific cancer incidence and combining HDR and LDR brachytherapy, utilization trends showed decreases in Alberta and British Columbia, increases in Ontario, Nova Scotia, and Saskatchewan, and no significant change in Manitoba, Québec, or nationally.

**Conclusions:**

This study revealed significant regional variations in brachytherapy utilization and clinical practices across Canadian radiotherapy centres. Factors influencing these trends include reimbursement structures, personnel and infrastructure availability, and clinician preferences. The use of HDR modalities is increasing nationally, while LDR is declining. Despite significant regional differences, brachytherapy utilization in Canada was largely sustained over the decade from 2011 to 2020.

## Highlights

• Brachytherapy use across twenty-five Canadian centres was evaluated over ten years.
• High-dose-rate treatments increased and became the dominant modality nationwide.
• Low-dose-rate prostate implants decreased in every reporting province since 2011.
• Incidence-adjusted trends showed significant regional variations across provinces.
• Local infrastructure and clinician preferences influenced national utilization rates.

## Graphical abstract

**Figure:**
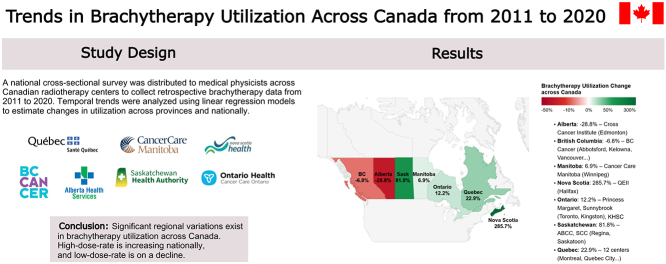

## 1 Introduction

Radiation oncology has been recognized as a specialty in Canada since the late 1940s [1]. Cancer care is largely funded by provincial governments, and standards of radiotherapy practice are established by professional associations of radiation oncologists (CARO), medical physicists (COMP), and medical radiation technologists (CAMRT) [2], [3]. Radiotherapy services are centralized within provincial cancer agencies that coordinate access and resource allocation. Training in brachytherapy in Canada occurs during radiation oncology residency and through additional Royal College certification pathways [1]. Physicians may obtain the Diploma in Brachytherapy through either a 12-month Area of Focused Competence (AFC) training program at an accredited university (currently the University of British Columbia, University of Calgary, and University of Toronto) or through a Practice Eligibility Route (PER) recognizing established clinical expertise [4].

The two main modalities of radiotherapy are external beam radiotherapy (EBRT) and brachytherapy, which are distinguished by whether the radiation source is located away from the patient’s body or implanted directly into or near the target volume. Brachytherapy is a highly conformal form of radiotherapy and one of the most cost-effective radiation treatments [5], [6]. In Canada, brachytherapy programs developed rapidly in the 1990s and are currently offered in approximately 60% of radiotherapy clinics nationwide [7]. For several oncological indications, such as cervical cancer and prostate cancer, brachytherapy combined with EBRT has been shown to provide superior survival outcomes compared with EBRT alone [8], [9], [10], [11], [12]. Despite this evidence, reports over the past decade have indicated a steady decline in brachytherapy utilization, particularly in the United States [13], [14], [15], [16], [17], [18]. In contrast, several recent studies examining cervical cancer treatments have reported an increase in brachytherapy utilization, accompanied by favorable clinical outcomes [10], [19].

A study by Rose et al. in 2013 suggested a mismatch between demand and availability of brachytherapy in Canada due to resource limitations across radiotherapy centres [20]. However, the study did not report the full longitudinal data of the number of treatments per centre and instead focused more on the indications treated, clinical workflow, and the professional experience of radiation oncologists. A more recent study by Lecavalier-Barsoum et al. reported a steady utilization of brachytherapy across twelve hospital centres in the province of Québec, from 2011 to 2019 [7]. Nevertheless, potential variations in clinical protocols and available resources could cause significant differences in brachytherapy practice in other regions of Canada. This study aimed to quantify changes in the utilization of brachytherapy for all relevant cancer types across Canada in a designated period from 2011 to 2020 and to identify potential reasons behind disparities across the country.

## 2 Materials and methods

### 2.1 Data collection

A cross-sectional survey was designed to investigate brachytherapy utilization in Canadian radiotherapy centres over the period from 2011 to 2020. The questionnaire was developed using the online platform SurveyHero [21] and distributed to medical physicists at Canadian radiotherapy centres offering brachytherapy. Of those distributed, thirteen surveys were fully completed. This included the following provinces and institutions:

• Alberta: Cross Cancer Institute (Edmonton);
• British Columbia: The BC Cancer Agency provided cumulative data across five regional centres^1^ (Abbotsford/Surrey, Kelowna, Prince George, Vancouver, and Victoria);
• Manitoba: Cancer Care Manitoba (CCMB, Winnipeg);
• Nova Scotia: QEII Cancer Centre (Halifax);
• Ontario: Kingston Health Sciences Centre (KHSC, Kingston), Princess Margaret Cancer Centre (Toronto), and Sunnybrook Health Sciences Centre (Toronto);
• Saskatchewan: Allan Blair Cancer Centre (Regina) and Saskatoon Cancer Centre (Saskatoon).

While brachytherapy is also offered in radiotherapy centres in New Brunswick and Newfoundland, the representatives in those provinces were unable to participate in the survey due to technical challenges in extracting data from local repositories. In addition, patients from Prince Edward Island who are prescribed brachytherapy treatments are referred out of province to Moncton or Halifax with an average of about five patients per year (based on private correspondence). Those are partially included in the QEII Cancer Centre dataset. For technical reasons, the QEII dataset reports the number of treated patients, whereas all other datasets quantify the number of fractions delivered for each modality. For Québec, we used the longitudinal dataset from 2011 to 2019 previously published by Lecavalier-Barsoum et al. [7] that was obtained from the Ministère de la Santé et des Services Sociaux du Québec. Because this unified dataset captures all reported brachytherapy procedures in Québec during the study period, the retrospective counts are fixed and not expected to change. Considering that the study did not report on treated oncological indications or other logistical details, a follow-up survey was distributed to twelve radiotherapy centres in Québec to record the types of cancers treated during the same period. Out of 39 radiotherapy centres conducting brachytherapy treatments in Canada from 2011 to 2020, the data in this study represent 25 of those, corresponding to ∼64% response rate.

The main questionnaire included questions on brachytherapy treatment modalities, as well as details on the annual number of delivered treatments (see Supplementary Material for the original survey form). The shorter Québec survey collected only qualitative data on brachytherapy modalities by treatment site. Ethical approval was not required because the survey collected institutional practice data without patient-specific information. In HDR brachytherapy, patients receive multiple radiation fractions; each fraction was counted as an individual treatment unless stated otherwise.

Brachytherapy modalities were categorized into high-dose-rate (HDR), low-dose-rate (LDR), and pulsed-dose-rate (PDR). Delivery techniques are further divided into subtypes based on the route of administration: intracavitary (placement of the radioactive source within a body cavity), interstitial (placement directly into tissue), plesiotherapy (surface application), and episcleral plaque (for ocular tumors). Commonly used subtypes include HDR-intracavitary (HDR IC), HDR-interstitial (HDR IT), HDR IC+IT, HDR-plesiotherapy (HDR Plesio), LDR-intracavitary (LDR IC), LDR-interstitial (LDR IT), LDR-eye plaque, PDR-intracavitary (PDR IC), PDR-interstitial (PDR IT), and PDR IC+IT.

**Fig. 1:**
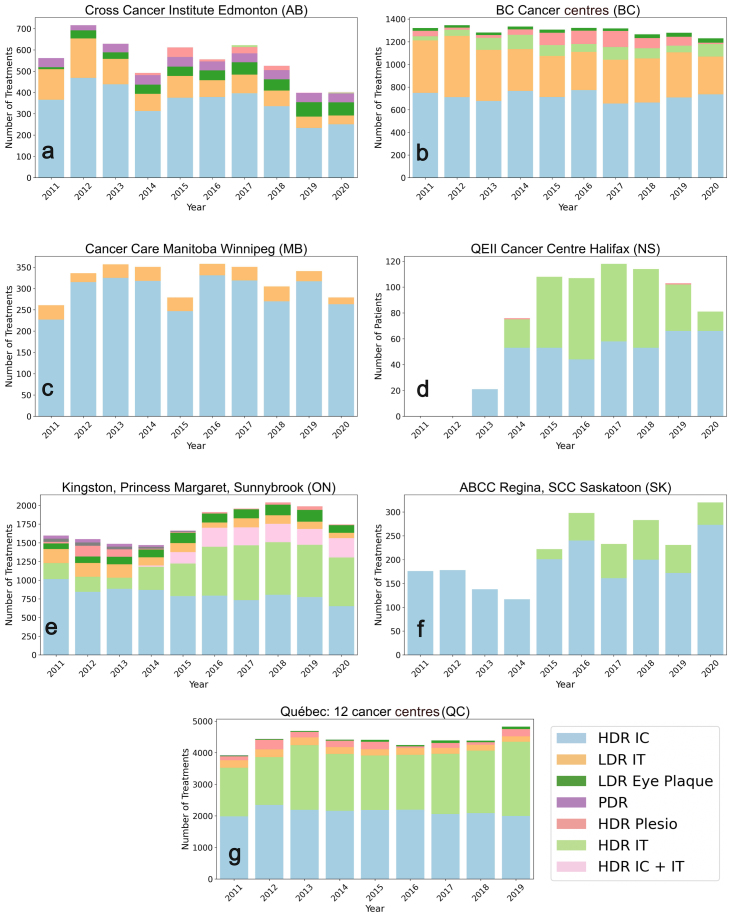
Trends in utilization of different brachytherapy modalities as reported by radiotherapy centres in Alberta (A), British Columbia (B), Manitoba (C), Nova Scotia (D), Ontario (E), Saskatchewan (F), and Québec (G) from 2011 to 2020. The data for Québec was taken from Lecavalier-Barsoum et al. [7].

### 2.2 Data analysis

The statistical analysis was performed using Microsoft Excel (Microsoft Corporation, Redmond, WA, USA) and R version 4.4.1 [22]. To account for changes in cancer incidence per province from 2011 to 2020, the incidence-adjusted brachytherapy utilization rate was calculated, as the change in the number of brachytherapy treatments normalized by the number of new brachytherapy-eligible cancer cases per province from 2011 to 2020 [23]. In particular, the survey data recorded which oncological indications are being treated with a particular brachytherapy modality for each radiotherapy centre, and so the corresponding temporal cancer incidence was extracted from the Statistics Canada dataset in each province. Cancer incidence-adjusted rates of change in brachytherapy utilization from 2011 to 2020 were estimated using linear regression, using the *lm* function from the *stats* R package. Fractions per 100 indication-specific incident cases of cancer as a function of year were modeled separately for HDR, LDR, and both HDR and LDR, for each province and for Canada, with no other covariates. Two-tailed 95% confidence intervals and corresponding p-values were calculated. Cancer incidence data were not available for Nova Scotia (2020) and Québec (2018–2019), so were imputed via linear extrapolation. Québec and Canada-wide estimates exclude the year 2020 as data were not available for Québec in 2020.

## 3 Results

### 3.1 Trends in brachytherapy utilization

The number of annual brachytherapy treatments per modality was obtained from 25 cancer centres across seven Canadian provinces. Fig. 1 shows the annual number of delivered treatments for each brachytherapy modality per province.^2^ HDR brachytherapy represented the dominant treatment modality throughout 2011–2020 across all participating centres, primarily driven by the intracavitary treatments for gynecological cancers. Several provinces reported 60%–90% of annual cases as HDR. Interstitial and plesiotherapy HDR modalities contributed smaller but stable fractions depending on the treated indications. In contrast, LDR usage was more variable and generally declined over time, particularly for prostate implants. LDR eye-plaque treatments remained a small but steady component in provinces where they were offered (Alberta, BC, Ontario, and Quebec). PDR brachytherapy was used only at the Cancer Institute in Edmonton and Princess Margaret in Toronto, and contributed minimally overall. The full explanation of trends for each modality per province is provided in the Supplementary Material Tables S1–S4.

Relative changes in HDR, LDR, and PDR usage across provinces during the study period are summarized in Table 1. Overall, HDR treatments increased in most centres over time, while Edmonton was the only centre showing a net decline. HDR activity remained relatively stable in British Columbia and Manitoba, whereas Ontario showed a clear increase associated with expanded HDR IT and hybrid IC+IT protocol implementation. The largest growth in HDR treatments occurred in Nova Scotia and Saskatchewan, reflecting the introduction and expansion of HDR IT programs during the study period. In contrast, LDR usage declined across all provinces. PDR treatments were limited to Edmonton, where usage remained stable, and Princess Margaret in Ontario, where the modality was decommissioned during the study period. At the national level, pooled data indicate an overall increase in HDR treatments accompanied by decreases in both LDR and PDR usage, resulting in a net increase in the total number of brachytherapy treatments.

**Fig. 2:**
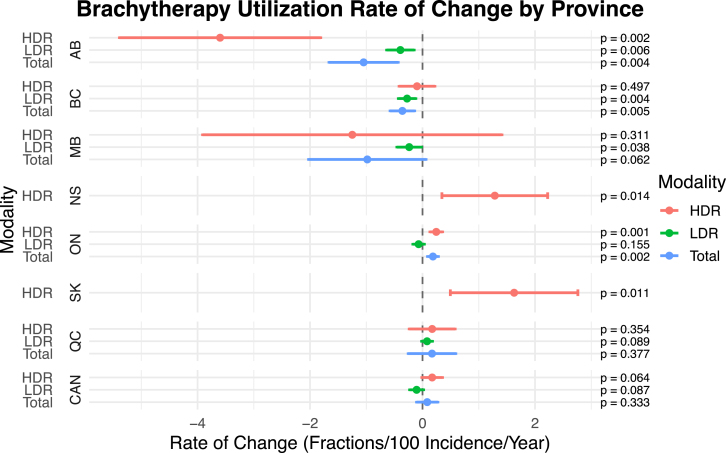
Incidence-adjusted HDR, LDR, and both HDR and LDR brachytherapy utilization rates of change per province, from 2011 to 2020. Estimates are based on linear regression, with 95% confidence intervals and p-values presented. Québec and Canada-wide estimates exclude the year 2020 as data were not available for Québec in 2020.

Incidence-adjusted trends in brachytherapy utilization from 2011–2020 are shown in Fig. 2. Estimates for Québec and Canada exclude 2020 due to unavailable Québec data; analyses including 2020 while excluding Québec yielded similar results. HDR utilization decreased in Alberta (−3.6 fractions per 100 indication-specific incident cases per year, 95% CI −5.39 to −1.81, p = 0.002) and increased in Nova Scotia (1.28, 95% CI 0.34 to 2.22, p = 0.014), Ontario (0.24, 95% CI 0.13 to 0.36, p = 0.001), and Saskatchewan (1.63, 95% CI 0.49 to 2.76, p = 0.011). No significant change was observed in British Columbia, Manitoba, Québec, or nationally. Only HDR data were available for Nova Scotia and Saskatchewan. LDR utilization decreased in Alberta (−0.39, 95% CI −0.64 to −0.15, p = 0.006), British Columbia (−0.27, 95% CI −0.43 to −0.12, p = 0.004), and Manitoba (−0.24, 95% CI −0.46 to −0.02, p = 0.038), with no significant change in Ontario, Québec, or nationally. Combined HDR+LDR utilization decreased in Alberta (−1.05, 95% CI −1.66 to −0.43, p = 0.004) and British Columbia (−0.36, 95% CI −0.58 to −0.14, p = 0.005), increased in Ontario (0.19, 95% CI 0.09 to 0.28, p = 0.002), and showed no significant change in Manitoba, Québec, or across Canada.

**Table:** Relative difference over various time periods comparing the utilization of brachytherapy per province. The relative change for different modalities is quoted with respect to the beginning of each period. The minimum and maximum annual change are also given in parentheses for each period.

Cross Cancer Institute Edmonton (AB)				
Period	HDR Total	LDR Total	PDR total	Total
2011–2015^a^	15.0 (−26.4, 30.3)	−4.6 (−32.7, 45.8)	4.7 (−44.2, 66.7)	8.9 (−21.8, 27.4)
2015–2020	−39.4 (−33.7, 11.8)	−29.5 (−14.9, 16.0)	−6.7 (−6.7, 2.4)	−34.6 (−23.8, 11.9)
2011–2020	−30.3 (−33.7, 30.3)	−32.7 (−32.7, 45.8)	−2.3 (−44.2, 66.7)	−28.8 (−23.8, 27.4)

BC Cancer centres (BC)				
Period	HDR Total	LDR Total	Total	
2011–2015	10.2 (−5.8, 16.2)	−20.1 (−16.7, 15.0)	−1.0 (−4.8, 4.1)	
2015–2020	−6.4 (−7.2, 4.7)	−4.6 (−14.1, 13.3)	−5.9 (−3.9, 1.1)	
2011–2020	3.1 (−7.2, 16.2)	−23.8 (−16.7, 15.0)	−6.8 (−4.8, 4.1)	

Cancer Care Manitoba (MB)				
Period	HDR IC	LDR IT	Total	
2011–2015	8.8 (−22.3, 38.8)	−5.9 (−38.2, 52.4)	6.9 (−20.5, 28.7)	
2015–2020	6.5 (−17.0, 34.0)	−50.0 (−33.3, 18.5)	0 (−18.2, 28.3)	
2011–2020	15.9 (−22.3, 38.8)	−52.9 (−38.2, 52.4)	6.9 (−20.5, 28.7)	

QEII Cancer Centre (NS)				
Period	HDR IC	HDR IT^b^	HDR Total	
2015–2020	24.5 (−17, 31.8)	−72.7 (−58.3, 14.5)	−25.0 (−21.4, 10.3)	
2013–2020	214.3 (−17.0, 152.4)	−31.8 (−58.3, 150.0)	285.7 (−21.4, 261.9)	

Princess Margaret, KHSC, Sunnybrook (ON)				
Period	HDR Total	LDR Total	PDR Total	Total
2011–2015	10.9 (−4.9, 14.9)	−2.3 (−23.8, 19.6)	−76.2 (−60.0, 4.8)	6.3 (−3.7, 14.3)
2015–2020	13.4 (−9.4, 24.1)	−32.8 (−32.0, 27.4)	NA	5.6 (−12.3, 15.6)
2011–2020	25.7 (−9.4, 24.1)	−34.4 (−32.0, 27.4)	NA	12.2 (−12.3, 15.6)

ABCC Regina and SCC Saskatoon (SK)				
Period	HDR IC	HDR IT	HDR Total	
2011–2015	14.2 (−22.5, 71.8)	NA	26.1 (−22.5, 89.7)	
2015–2020	35.8 (−32.9, 58.7)	123.8 (−28.9, 176.2)	44.1 (−21.8, 38.5)	
2011–2020	55.1 (−32.9, 71.8)	NA	81.8 (−22.5, 89.7)	

Québec (12 radiotherapy centres [7])				
Period	HDR Total	LDR Total	Total	
2011–2015	13.7 (−5.8, 14.1)	−6.4 (−8.0, 3.2)	12.3 (−5.9, 13.0)	
2015–2019	10.4 (−3.9, 10.4)	−5.3 (−10.6, 5.1)	9.4 (−3.8, 10.2)	
2011–2019	25.5 (−5.8, 14.1)	−11.4 (−10.6, 5.1)	22.9 (−5.9, 13.0)	

Canada				
Period	HDR Total	LDR Total	PDR total	Total
2011–2015	14.6 (−1.9, 9.2)	−10.8 (−15.9, 11.2)	−35.3 (−21.4, 11.8)	10.1 (−3.9, 9.2)
2015–2019	8.0 (−0.7, 4.6)	−0.6 (−11.7, 13.9)	−21.8 (−23.6, 2.4)	6.7 (−0.8, 2.9)
2011–2019	23.8 (−1.9, 9.2)	−11.3 (−15.9, 13.9)	−49.4 (−23.6, 11.8)	17.5 (−3.9, 9.2)

a Inclusive with 2015.

b Data reported from 2014.

**Fig. 3:**
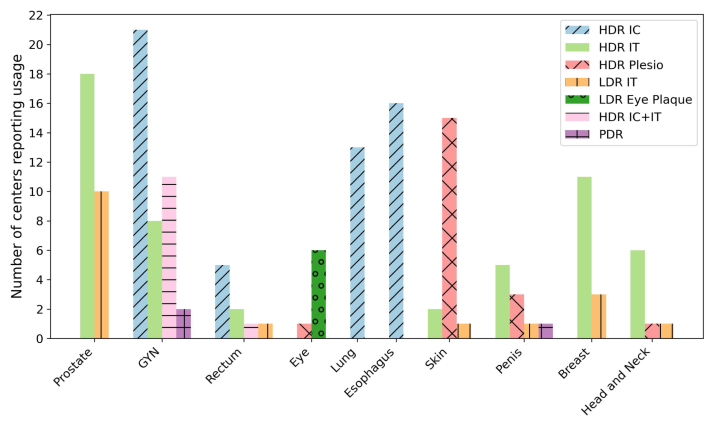
The number of radiotherapy centres reporting the usage of a particular brachytherapy modality for treating the following indications: prostate, gynecological sites (GYN), rectum, eye, lung, esophagus, skin, penis, breast, and head and neck cancers. The data corresponds to the survey period from 2011 to 2020 and includes twenty-five radiotherapy centres across Canada.

### 3.2 Variety of treated oncological indications

Brachytherapy is most frequently used for prostate and gynecological (GYN) indications, with the widest range of treatment techniques applied to GYN sites (Fig. 3). Prostate cancers are treated using HDR IT and LDR IT techniques. GYN indications are treated using HDR IC, hybrid IC+IT techniques, HDR IT, and PDR. HDR IC is additionally used for lung, esophagus, and rectal cancers. HDR IT and IC techniques were also applied to penile, breast, and head and neck cancers during the survey period. Rectal cancer patients are treated using HDR IT, and at Sunnybrook a hybrid IC+IT technique is offered to a selected group of patients. Eye indications are treated primarily using plaque-based LDR techniques. Several centres use HDR plesiotherapy for skin cancers and occasionally for penile and head and neck cancers. Princess Margaret and Kingston also report the use of HDR IT for sarcoma treatments. During the survey period, several centres reported discontinuing specific brachytherapy protocols in favor of external beam treatments. A more detailed summary of treated oncological indications per province is provided in the Supplementary Material Tables S5–S7.

## 4 Discussion

The study identified regional disparities in brachytherapy utilization across Canada from 2011 to 2020. During the survey period, brachytherapy was used to treat a range of clinical indications, mostly gynecological and prostate cancer, but also rectal, ocular, pulmonary, esophageal, dermatological, penile, breast, sarcoma, and head and neck cancers. The total raw number of brachytherapy treatments increased at radiotherapy centres in Manitoba, Ontario, Saskatchewan, Québec and Nova Scotia, while it declined in BC, and most significantly in Alberta. During that period, HDR represented 60%–95% of all brachytherapy treatments and showed an increase from 3%–80% from 2011 to 2020 in all provinces except Alberta where it decreased by 30%. LDR treatments used in Alberta, BC, Manitoba, Ontario, and Québec showed a decline of 10%–50% during the survey period. Incidence-adjusted regression analysis showed declining HDR utilization in Alberta and increasing utilization in Nova Scotia, Ontario, and Saskatchewan, with no significant changes observed in the remaining provinces or nationally. For LDR modalities, utilization declined in Alberta, British Columbia, and Manitoba, with no evidence of change in Ontario, Québec, or at the national level. After combining HDR and LDR data, regression-based brachytherapy utilization appeared to be sustained across Canada.

An observed decline in LDR IT utilization during the survey period is attributed mainly to protocol changes in prostate cancer treatments. Some centres reported increasing adoption of IMRT/SBRT referrals for low- and favorable-intermediate-risk prostate cancer patients, accompanied by frequent challenges related to LDR safety requirements. Another reason was the availability and increase in HDR referrals in accordance with emerging clinical evidence suggesting lower toxicity compared to LDR prostate cancer brachytherapy [24], [25], [26], [27].

Possible reasons for disparities between centres include infrastructure availability, workforce training, absence of local expertise (e.g., for LDR eye-plaque treatments), and clinician preference for alternative modalities such as EBRT or SBRT. Larger centres showed more stable brachytherapy use, while smaller centres experienced greater variation due to protocol changes or personnel turnover. During the study period, various radiotherapy centres reported significant changes in brachytherapy protocols. Several centres reduced the number of fractions for gynecological HDR treatments following GEC-ESTRO protocol updates and COVID-19 pressures. A reduced rate of LDR IT treatments was in general attributed to fewer surgical referrals for prostate cancer patients who were instead treated with SBRT and VMAT protocols. In some Québec centres, there was an initiative of replacing LDR IT with HDR IT protocols.

Historically, brachytherapy utilization in the United States was reported to be in decline, particularly for prostate cancer during the early 2000s [13], [14], [15], [16]. Despite the continuous declining trends for prostate cancer [28], more recent analyses focusing on the period after 2010 suggested a stabilization or an increase in utilization for gynecological cancers [10], [19], [29]. While our findings in Canada show a sustained national utilization, the US landscape reflects a complex interplay of factors. The emergence of competing technologies, such as SBRT, has impacted brachytherapy’s market share [28], while financial pressures and Medicare reimbursement have also influenced practice trends [10], [17], [18]. In addition, the training landscape presents a major point of difference between the two healthcare systems. In Canada, the Royal College of Physicians and Surgeons has formalized expertise through a dedicated “Area of Focused Competence” diploma, providing a standardized certification pathway [1]. In contrast, the US model relies on numerical residency case-log minimums, which have seen a documented decline in interstitial and prostate procedures, leading to a potential competency gap for new practitioners [28], [30]. These multifaceted factors — ranging from technological competition and financial incentives to specialized educational frameworks — collectively shape the evolving utilization trends observed in both regions.

### 4.1 Methodological limitations and future directions

With the data presented across a variety of brachytherapy centres in Canada, this study has several limitations. First, the datasets are heterogeneous, as each Canadian province maintains a distinct system for recording radiotherapy treatments, in addition to centre-specific databases that the individual authors were required to consult to compile longitudinal brachytherapy data. Furthermore, while the study contains the full dataset for provinces of British Columbia, Manitoba, Nova Scotia, Saskatchewan, and Québec, we were not able to obtain the data from Calgary, as a major radiotherapy centre in Alberta, or any data from New Brunswick or Newfoundland. In a similar way, smaller centres are underrepresented in Ontario which, according to the Canadian 2021 census, correspond to 46% of the total Ontario population. Based on the population served by the brachytherapy centres included in the study, we estimate that the dataset captures approximately 72% of all brachytherapy treatments nationally. The unrepresented data could, in principle, exhibit a different trend based on provincial decisions. Second, incidence-adjusted brachytherapy utilization rates were estimated using provincial incidence data, as the survey did not include patient-level variables such as cancer type, age, or stage, and instead comprised cumulative retrospective data. Despite this limitation, particular consistent trends across regions suggest that the shift in modality, such as the transition from LDR to HDR, reflects systemic changes in infrastructure and health policy rather than differences in patient demographics. Future work incorporating patient-level data will enable more robust multivariable analyses. Third, centre-dependent changes in protocols, such as decreasing the number of fractions, may result in an apparent decrease in HDR utilization, which does not indicate an authentic decrease in its use. Additionally, non-quantifiable factors such as reimbursement policies and radiation oncologists’ preferences may also impact the variation in brachytherapy utilization at individual cancer centres. Next, regression-based estimates of changes in brachytherapy usage across time assumed linear trends, ignoring potential non-linear relationships. Finally, the study relies on multiple data sources, including local hospital databases and government reimbursement forms, in a non-standardized way. Creating a standardized database for brachytherapy treatments across Canada could be a subject of future efforts.

Improving and expanding brachytherapy services in Canada depends on addressing the limitations caused by various logistical and financial factors. Brachytherapy has a labor-intensive nature, requiring specialized radiation oncologists, medical physicists, radiation therapists, and technologists, as well as a time-consuming treatment planning and delivery process. However, the impact of improved survival and quality of life for suitable cancer patients may outweigh its human, financial, and time costs [15], [17]. Artificial intelligence-driven automation of brachytherapy treatment planning [31], [32], [33] may improve workflow efficiency through automated segmentation, catheter reconstruction, and treatment optimization [34], [35], [36], [37], [38], allowing clinicians to focus more on direct patient care. Sufficient government funding and a fair referral mechanism are pertinent to achieving equitable access to brachytherapy. This requires the optimization of resource allocations and the expansion of brachytherapy facilities to meet patient demands in each region.

## CRediT authorship contribution statement

**Mirta Dumančić:** Writing – review & editing, Writing – original draft, Visualization, Methodology, Investigation, Funding acquisition, Formal analysis, Data curation, Conceptualization. **Yujing Zou:** Writing – review & editing, Writing – original draft, Visualization, Validation, Methodology, Formal analysis, Data curation, Conceptualization. **Harry Glickman:** Writing – review & editing, Visualization, Software, Methodology, Formal analysis, Data curation. **Sara Ghassimi Kouraneh:** Formal analysis, Data curation. **Piotr Pater:** Writing – review & editing, Visualization, Methodology, Data curation. **Andrew Alexander:** Writing – review & editing, Data curation. **Jorge E. Alpuche Aviles:** Writing – review & editing, Data curation. **Amanda Cherpak:** Writing – review & editing, Data curation. **Kristin Marchant:** Writing – review & editing, Data curation. **Geetha Menon:** Writing – review & editing, Data curation. **Daniel Morton:** Writing – review & editing, Data curation. **Tim Olding:** Writing – review & editing, Data curation. **Moti Paudel:** Writing – review & editing, Data curation. **Alexandra Rink:** Writing – review & editing, Data curation. **Ingrid Spadinger:** Writing – review & editing, Data curation. **Shirin A. Enger:** Writing – review & editing, Supervision, Resources, Project administration, Methodology, Investigation, Funding acquisition, Conceptualization.

## Declaration of competing interest

The authors declare that they have no known competing financial interests or personal relationships that could have appeared to influence the work reported in this paper.

## Appendix A Supplementary data

MMC S1 Supplementary tables detailing brachytherapy modalities, indications, and treatment trends across Canadian centres (2011–2020). MMC S1

## Footnotes

1 Surrey patients are treated at Abbotsford Cancer Centre and included in that dataset.

## Footnotes

2 LDR and PDR data represent the number of unique patients since no fractionation schedule is applied in the clinical protocol.
