# Supplementary material for: Trends in brachytherapy utilization in Canada from 2011 to 2020
Source: Phys Imaging Radiat Oncol. 2026 Apr 30;39:100974. doi: 10.1016/j.phro.2026.100974 (PMC13157207; doi:10.1016/j.phro.2026.100974)
Supplement: MMC S1 — Supplementary tables detailing brachytherapy modalities, indications, and treatment trends across Canadian centres (2011–2020). [file mmc1.pdf]

Supplementary Material

Trends in brachytherapy utilization in Canada from 2011 to 2020

Contents

- Table S1. Annual number of brachytherapy treatments per modality and province.
- Table S2. Number of brachytherapy treatments per modality subtype delivered between 2011 and 2020 in Alberta.
- Table S3. Number of brachytherapy treatments per modality subtype delivered between 2011 and 2020 in British Columbia.
- Table S4. Number of treatments per modality subtype delivered between 2011 and 2020 at Princess Margaret, Kingston Health Sciences Centre, and Sunnybrook in Ontario.
- Table S5. Available brachytherapy modalities for different clinical indications in various provinces across Canada.
- Table S6. Summary of brachytherapy treatments offered for a variety of oncological indications in centres across Canada (except Québec).
- Table S7. Summary of brachytherapy treatments offered for a variety of oncological indications in centres across Québec.
- Survey form used in this study.

1. Supplementary tables

Table S1: Number of delivered brachytherapy treatments between 2011 and 2020 in Alberta (Cross Cancer Institute in Edmonton), British Columbia (BC Cancer Abbotsford, Kelowna, Prince George, Vancouver, and Victoria), Manitoba (Cancer Care Manitoba Winnipeg), Nova Scotia (QEII Cancer Centre Halifax), Ontario (Princess Margaret and Sunnybrook in Toronto and KHSC in Kingston), and Saskatchewan (ABCC Regina and SCC Saskatoon). The data for Québec are taken from Lecavalier-Barsoum *et al.* between 2011 and 2019. The treatments are divided into HDR, LDR, and PDR, and the total number per year. IT: interstitial, IC: intra-cavitary.

**Number of brachytherapy treatments per province**				
**Cross Cancer Institute Edmonton (Alberta)**				
Year	HDR Total (% fraction)	LDR Total (% fraction)	PDR Total (% fraction)	Total
2011	366 (66.2%)	153 (27.2%)	43 (7.8%)	553
2012	469 (69.2%)	223 (31.1%)	24 (3.5%)	678
2013	439 (73.4%)	150 (23.8%)	40 (6.7%)	598
2014	323 (71.9%)	124 (25.2%)	45 (10.0%)	449
2015	421 (74.1%)	146 (23.9%)	45 (7.9%)	568
2016	389 (76.3%)	125 (22.5%)	42 (8.2%)	510
2017	435 (77.1%)	145 (23.3%)	42 (7.4%)	564

2018	356 (75.4%)	126 (24.0%)	43 (9.1%)	472
2019	236 (71.1%)	121 (30.3%)	43 (13.0%)	332
2020	255 (75.4%)	103 (25.8%)	42 (12.4%)	338

BC Cancer centres (BC)
Abbotsford, Kelowna, Prince George, Vancouver, and Victoria

Year	HDR Total (% fraction)	LDR Total (% fraction)	Total
2011	833 (63.1%)	488 (36.9%)	1321
2012	785 (58.3%)	561 (41.7%)	1346
2013	809 (63.1%)	473 (36.9%)	1282
2014	940 (70.5%)	394 (29.5%)	1334
2015	918 (70.2%)	390 (29.8%)	1308
2016	961 (72.7%)	361 (27.3%)	1322
2017	909 (69.0%)	409 (31.0%)	1318
2018	844 (66.7%)	422 (33.3%)	1266
2019	847 (66.2%)	433 (33.8%)	1280
2020	859 (69.8%)	372 (30.2%)	1231

Cancer Care Manitoba Winnipeg (Manitoba)

Year	HDR IC (% fraction)	LDR IT (% fraction)	Total
2011	227 (87.0%)	34 (13.0%)	261
2012	315 (93.8%)	21 (6.3%)	336
2013	325 (91.0%)	32 (9.0%)	357
2014	318 (90.6%)	33 (9.4%)	351
2015	247 (88.5%)	32 (11.5%)	279
2016	331 (92.5%)	27 (7.5%)	358
2017	319 (90.9%)	32 (9.1%)	351
2018	270 (88.5%)	35 (11.5%)	305
2019	317 (93.0%)	24 (7.0%)	341
2020	263 (94.3%)	16 (5.7%)	279

QEII Cancer Centre Halifax [1] (Nova Scotia)

Year	HDR IC (% fraction)	HDR IT (% fraction)	HDR Plesio	Total
2013	21 (100%)	0	0	21
2014	53 (69.7%)	22 (28.9%)	1 (1.3%)	76
2015	53 (49.1%)	55 (50.9%)	0	108
2016	44 (41.1%)	63 (58.9%)	0	107
2017	58 (49.9%)	60 (50.8%)	0	118
2018	53 (46.5%)	61 (53.5%)	0	114
2019	66 (64.1%)	36 (34.9%)	1 (1.0%)	103
2020	66 (81.5%)	15 (18.5%)	0	81

Princess Margaret, KHSC and Sunnybrook (Ontario)

Year	HDR Total (% fraction)	LDR Total (% fraction)	PDR Total (% fraction)	Total

[1] This dataset represents the number of unique patients and not the number of HDR treatments.

Number of treatments per province (continued)

2011	1251 (80.5%)	261 (16.8%)	42 (2.7%)	1555
2012	1191 (79.1%)	270 (17.9%)	44 (2.9%)	1505
2013	1133 (78.1%)	281 (19.4%)	36 (2.5%)	1450
2014	1207 (83.5%)	214 (14.8%)	25 (1.7%)	1446
2015	1387 (83.9%)	256 (15.5%)	10 (0.6%)	1653
2016	1721 (90.1%)	190 (9.9%)	0	1911
2017	1716 (87.6%)	242 (12.4%)	0	1958
2018	1784 (87.5%)	256 (12.5%)	0	2040
2019	1736 (87.3%)	253 (12.7%)	0	1989
2020	1573 (90.1%)	172 (9.9%)	0	1745

ABCC Regina and SCC Saskatoon (Saskatchewan)

Year	HDR IC (% fraction)	HDR IT (% fraction)	Total
2011	176 (100%)	0	176
2012	178 (100%)	0	178
2013	138 (100%)	0	138
2014	117 (100%)	0	117
2015	201 (90.5%)	21 (9.5%)	222
2016	240 (80.5%)	58 (19.5%)	298
2017	161 (69.1%)	72 (30.9%)	233
2018	200 (70.7%)	83 (29.3%)	283
2019	172 (74.5%)	59 (25.5%)	231
2020	273 (85.3%)	47 (14.7%)	320

Québec (12 radiotherapy centres)

Year	HDR Total (% fraction)	LDR Total (% fraction)	Total
2011	3650 (92.9%)	280 (7.1%)	3930
2012	4164 (93.7%)	278 (6.3%)	4442
2013	4419 (94.1%)	276 (5.9%)	4695
2014	4164 (94.3%)	254 (5.7%)	4418
2015	4151 (94.1%)	262 (5.9%)	4413
2016	3991 (94.0%)	256 (6.0%)	4247
2017	4127 (94.0%)	264 (6.0%)	4391
2018	4148 (94.6%)	236 (5.4%)	4384
2019	4581 (94.9%)	248 (5.1%)	4829

In Edmonton, Alberta, the number of administered treatment modalities is diverse, with the majority administered by HDR IC, accounting for 66%-74% of all annual treatments. Those were mostly vaginal vault brachytherapy (prescribed in 3 or 5 fractions). HDR plesio is used for skin lesions, with a typical 10 fractions per patient treatment regimen. LDR fraction was recorded at the rate of 23-31% of all annual treatments, administered for prostate and eye plaque indications. A steady decline in LDR IT use for prostate seed implants has been recorded between 2012 and 2020, with an overall decrease of 72%. PDR treatments showed an increase from 8% of all treatments in 2011 to 12.4% in 2020, with an increase in PDR IC+IT treatments from 2017. These were mostly used for patients with cervical cancer and

other gynecological indications.

In British Columbia, 60-70% of all reported treatments are HDR, and the rest are LDR, depending on the year. The number of cases for HDR and LDR are stable overall, with yearly fluctuations around 10%. Most cases are treated with HDR IC (median annual number 712), followed by LDR IT (median 388). For this dataset, HDR IC+IT was categorized as either HDR IT or HDR IC, as there was no separate category in the database used for data extraction. HDR IT and HDR Plesio steadily contribute below 150 treatments per year. Finally, LDR eye plaque accounts for approximately 21 to 39 treatments annually.

At CCMB Winnipeg, Manitoba, 87-94% of annual treatments were HDR IC for gynecological cancers administered in 3-4 fractions. A steady contribution of 6-13% annual treatments was LDR IT used for prostate cancers. In Nova Scotia, the QEII centre reported the data exclusively for HDR modalities from 2013 to 2020. Due to technical circumstances, the numbers shown represent the number of treated patients and not the number of treatments. The data shows an overall increase in HDR IC usage for gynecological cancers, accounting for 41-81% of all treated patients. HDR IT is used for gynecological and prostate cancers, with the prostate protocol being only a single-fraction treatment. Since 2013, treatments increased, stabilizing at ∼60 patients annually from 2015 to 2018, before dropping to 15 in 2020.

In Ontario, Kingston centre reports only HDR modalities, while Princess Margaret and Sunnybrook also use LDR. The majority of treatments were HDR IC with a fraction of ∼55% up to 2014, after which an increase in HDR IT and a hybrid IC+IT approach was recorded. HDR represented 78-90% of annual treatments in that period. LDR eye plaque treatments conducted at the Princess Margaret represented a consistent contribution of 14-24% of all local treatments. LDR IT was on a constant decline at Princess Margaret, from 17% of all treatments in 2011 to only 3% in 2020. At Sunnybrook, on the other hand, a similar trend lasted until 2016 (from 100 to 70 patients per year), after which more patients were recruited for LDR IT, presumably due to the PR19 clinical trial [**?**]. PDR modalities were conducted only at Princess Margaret, which were 1-8% of all local treatments until 2015, after which the protocol was fully decommissioned.

In Saskatchewan, 69-90% of all annual treatments were administered with HDR IC for gynecological cancers (3-5 fractions), and 10-30% with HDR IT for prostate cancers (single fraction). The combined number of HDR IC treatments shows an overall increase of 55% from 2011 to 2020. A steady rise in prostate HDR IT treatments was reported in Regina, from 21 cases in 2015 to 83 cases in 2018. A ∼30% drop is observed in 2019 compared to the previous year, and an additional 20% in 2020. In Québec, for completeness, it also shows the fraction of annual treatments per modality as reported by Lecavalier-Barsoum *et al.*. HDR IC contributes a steady fraction from 41% to 53% of all treatments, followed by HDR IT from 34%-49%. Only 4-6% of all annual treatments are LDR IT. A smaller fraction, about 0.5-2% of treatments, are LDR-eye plaque. No PDR treatments were reported from 2011 to 2020 in Québec.

Brachytherapy treatments at Cross Cancer Institute in Edmonton (Alberta)

Year	HDR IC	HDR IT	HDR IC+IT	HDR Plesio	LDR IT	LDR Eye Plaque	PDR IC	PDR IT	PDR IC+IT
2011	366	0	0	0	144	9	41	0	2
2012	469	0	0	0	185	38	24	0	0
2013	439	0	0	0	119	31	39	0	1
2014	313	0	0	10	81	43	43	0	2
2015	376	0	0	45	102	44	32	0	13
2016	379	0	0	10	79	46	27	0	15
2017	397	8	0	30	87	58	14	0	28
2018	336	0	1	20	73	53	5	3	35
2019	234	0	3	0	53	68	9	2	32
2020	251	4	3	0	41	62	8	0	34

Table S2: Number of brachytherapy treatments per modality subtype delivered between 2011 and 2020 in Alberta. Data reported from the Cross Cancer Institute in Edmonton, Alberta.

Brachytherapy treatments in British Columbia

Year	HDR IC	HDR IT	HDR Plesio	LDR IT	LDR Eye Plaque
2011	749	36	48	464	24
2012	712	54	19	540	21
2013	678	106	25	451	22
2014	766	126	48	370	24
2015	713	96	109	362	28
2016	774	70	117	337	24
2017	655	112	142	387	22
2018	664	90	90	389	33
2019	709	59	79	398	35
2020	736	112	11	333	39

Table S3: Number of brachytherapy treatments per modality subtype delivered between 2011 and 2020 in British Columbia (BC Cancer centres). Hybrid HDR IC+IT treatments were not categorized separately and are included primarily in the HDR IC numbers.

Brachytherapy treatments in Ontario

Year	HDR IC	HDR IT	HDR IC+IT	HDR Plesio	LDR IT	LDR-Eye Plaque	PDR
2011	1015	214	0	22	188	74	42
2012	844	202	0	145	185	85	44
2013	884	149	0	100	180	101	36
2014	870	309	18	10	110	104	25
2015	788	435	154	10	119	137	10
2016	793	655	253	20	71	119	0
2017	734	733	239	10	122	120	0
2018	805	702	247	30	114	142	0
2019	774	699	213	50	96	157	0
2020	653	651	259	10	71	101	0

Table S4: Number of treatments per modality subtype delivered between 2011 and 2020 at at Princess Margaret, KHSC, and Sunnybrook in Ontario.

Brachytherapy modality	Indications treated (province)
HDR IC	GYN (all), Rectum (ON, QC), Lung (BC, ON, QC), Esophagus (BC, ON, QC)
HDR IT	Prostate (BC, NS, SK, ON, QC), GYN (AB, BC, NS, ON, QC), Rectum (ON), Skin (QC), Penis (BC,QC), Breast (BC, ON, QC), Head and Neck (AB, ON, QC), Sarcoma (ON)
HDR IC+IT	GYN (AB, BC, NS, ON, QC), Rectum (ON)
HDR Plesio	Eye (BC[a]), Skin (AB, BC, NS, QC), Penis (BC), Head and Neck (QC, ON)
LDR IT	Prostate (AB, BC, MB, ON, QC), Rectum (QC), Skin (QC), Penis (QC), Breast (BC), Head and Neck (BC)
LDR Eye Plaque	Eye (AB, BC, ON, QC)
PDR[b]	GYN (AB, ON, decommissioned in 2015), Penis (ON, decommissioned in 2015)

Table S5: Available brachytherapy modalities for different clinical indications in various provinces across Canada. Provinces where the treatments have been reported are indicated in parentheses for each indication.

[a]These treatments at BC Cancer in Vancouver were performed with a ^{90}Sr applicator, instead of a regular HDR afterloader.

[b]PDR treatments were mostly decommissioned, except in Edmonton, Alberta.

Name of the Centre	City (Province)	Treatments and indications
Cross Cancer Institute	Edmonton (AB)	HDR IC: GYN, HDR IT: GYN, HDR IC+IT: GYN, HDR Plesio: Skin, LDR IT: Prostate, LDR Eye Plaque, PDR: GYN
BC Cancer Abbotsford	Abbotsford (BC)	HDR IC: GYN, Lung, Esophagus, HDR Plesio: Skin, LDR IT: Prostate
BC Cancer Kelowna	Kelowna (BC)	HDR IC: GYN, HDR IT: Prostate, GYN, Penis, Breast, HDR Plesio: Skin, Penis, LDR IT: Prostate, Breast
BC Cancer Prince George	Prince George (BC)	HDR IC: GYN
BC Cancer Vancouver	Vancouver (BC)	HDR IC: GYN, Lung, Esophagus, HDR IT: GYN, Penis, HDR Plesio: Eye, Skin, Penis, LDR IT: Prostate, Head and Neck, LDR Eye Plaque
BC Cancer Victoria	Victoria (BC)	HDR IC: GYN, Esophagus, HDR IT: GYN, LDR IT: Prostate, Breast
Cancer Care Manitoba	Winnipeg (MB)	HDR IC: GYN, LDR IT: Prostate
QEII Cancer Centre	Halifax (NS)	HDR IC: GYN, HDR IT: Prostate, GYN, HDR IC+IT: GYN, HDR Plesio: Skin
Kingston Health Sciences Centre	Kingston (ON)	HDR IC: GYN, Lung, Esophagus, HDR IT: Breast, Head and Neck, Keloid
Princess Margaret Cancer Centre	Toronto (ON)	HDR IC: GYN, Rectum, Lung, Esophagus, HDR IT: Prostate, GYN, Rectum, Head and Neck, HDR IC+IT: GYN, LDR IT: Prostate, LDR Eye Plaque, PDR: GYN, Penis
Sunnybrook Health Sciences Centre	Toronto (ON)	HDR IC: GYN, Rectum, Lung, Esophagus, HDR IT: Prostate, GYN, Rectum, HDR IC+IT: GYN, Rectum HDR Plesio: Skin, LDR IT: Prostate
Allan Blair Cancer Centre	Regina (SK)	HDR IC: GYN, HDR IT: Prostate
Saskatoon Cancer Centre	Saskatoon (SK)	HDR IC: GYN

Table S6: The full summary of brachytherapy treatments offered for a variety of oncological indications in centres across Canada (except Québec).

Name of the Centre	City	Treatments and indications
CISSS de l'Outaouais	Gatineau	HDR IC: Lung, Esophagus, HDR IT: Prostate, Skin, Breast, HDR IC+IT: GYN, HDR Plesio: Skin, LDR IT: Rectum, Skin, Penis
CISSS Laval	Laval	HDR IC: GYN, HDR IT: Prostate, LDR Eye Plaque
CISSS de Chaudière-Appalaches[a]	Lévis	HDR IC: GYN, Lung, HDR IT: Prostate, HDR IC+IT: GYN, LDR Eye Plaque
CISSS de la Montérégie-Centre (Hopital Charles LeMoyne)	Longueuil	HDR IC: Lung, Esophagus, HDR IT: Penis, Breast, Head and Neck HDR IC+IT: GYN, HDR Plesio: Skin, Head and Neck
Centre universitaire de santé McGill (McGill University Health Centre)	Montréal	HDR IC: Esophagus, HDR IT: Prostate, Penis, Breast, Head and Neck, HDR IC+IT: GYN, HDR Plesio: Skin
CIUSSS de l'Est-de-Montréal (Hopital Maisonneuve Rosemont)	Montréal	HDR IC: GYN, HDR IT: Prostate, Breast, HDR IC+IT: GYN, HDR Plesio: Skin
CIUSSS du Centre-Ouest-de-l'Île-de-Montréal (Jewish General Hospital)	Montréal	HDR IC: Rectum, Esophagus, HDR IT: Prostate, Penis, Breast, Head and Neck HDR IC+IT: GYN, HDR Plesio: Skin
Centre hospitalier de l'Université de Montréal (CHUM)	Montréal	HDR IC: GYN, Rectum, Lung, Esophagus, HDR IT: Prostate, Breast, Head and Neck HDR IC+IT: GYN, HDR Plesio: Skin, LDR IT: Prostate, LDR Eye Plaque
CISSS du Bas-Saint-Laurent	Rimouski	HDR IC: GYN, Lung, Esophagus, HDR IT: Prostate, Breast HDR Plesio: Skin
CIUSSS du Saguenay–Lac-Saint-Jean	Shawinigan	HDR IC: GYN, Lung, Esophagus, HDR IT: Prostate, Skin, Breast, HDR Plesio: Skin
Centre hospitalier universitaire de Sherbrooke	Sherbrooke	HDR IC: GYN, Lung, Esophagus, HDR IT: Prostate
CIUSSS de la Mauricie-et-du-Centre-du-Québec (MCQ)	Trois-Rivières	HDR IC: GYN, Lung, Esophagus, HDR IT: Prostate, Breast, Head and Neck HDR Plesio: Skin
Centre hospitalier universitaire de Québec–Université Laval	Québec City	HDR IC: GYN, Lung, Esophagus, HDR IT: Prostate, GYN, HDR IC+IT: GYN, LDR IT: Prostate, LDR Eye Plaque

Table S7: The full summary of brachytherapy treatments offered for a variety of oncological indications in centres across Québec. Fr. CISSS = Centre intégré de santé et de services sociaux; CIUSSS = Centre intégré universitaire de santé et de services sociaux.

[a] The centre in Lévis opened in 2019 and brachytherapy treatments started in 2020.

2. Survey form

The survey questions were organized into nine primary items, covering: (1) province of clinic location, (2) reimbursement structure, (3) brachytherapy usage, (4) reasons for non-use (if applicable), (5) annual number of brachytherapy treatments categorized by technique, (6) clinical indications for each treatment type, (7) consistency of treatment code usage, (8) listing of specific billing or tracking codes, (9) details of any changes in coding over the study period, and finally, (10) the number of treatments (or fractions) given per patient. In (5), the number of treatments represents each fraction of brachytherapy delivered.

Questionnaire Regarding Utilization of Brachytherapy in Canada

Questionnaire Regarding Utilization of Brachytherapy in Canada

Please enter your contact details:

First and last name

Email address

1. Which province is your clinic located in?

- ◯ Alberta
- ◯ British Columbia
- ◯ Manitoba
- ◯ New Brunswick
- ◯ Newfoundland and Labrador
- ◯ Northwest Territories
- ◯ Nova Scotia
- ◯ Nunavut
- ◯ Ontario
- ◯ Prince Edward Island
- ◯ Quebec
- ◯ Saskatchewan
- ◯ Yukon

2. Does your province self report to your provincial government to get reimbursement for radiation oncology treatments?

- ◯ Yes
- ◯ No

3. Did your province provide brachytherapy from beginning of 2011 to end of 2020 (please answer yes even if brachytherapy was offered only for part of this a period)

- ○ Yes
- ○ No

4. If the answer to question (3) is no, what is/are the reason/reasons?

You may choose more than one option

- ☐ Lack of trained personnel
- ☐ Lack of equipment
- ☐ Lack of patients
- ☐ Reimbursement
- ☐ Other

5. If the answer to question (3) is Yes, which of the following subtype of brachytherapy did your province offer?

Please report the number of unique patients treated with each modality.HDR refers to high dose rate. LDR refers to low dose rate. HDR IC refers to HDR intracavitary, HDR IT refers to HDR interstitial, HDR Plesio refers to HDR plesiotherapy, LDR IC refers to LDR-intracavitary, LDR IT refers to LDR interstitial.

Please fill in the number of treatments per years in the table below:

	HDR IC	HDR IT	HDR Plesio	LDR IC	LDR IT	LDR Eye plaque	HDR IC+IT
2011							
2012							
2013							
2014							
2015							
2016							
2017							

2018							
2019							
2020							

6. Please add the indication for each treatment type.

The columns are indications. You can select multiple options per row.

	HDR IC	HDR IT	HDR IC+IT	HDR Plesio	LDR IC	LDR IT	LDR Eye plaque
Prostate	☐	☐	☐	☐	☐	☐	☐
GYN	☐	☐	☐	☐	☐	☐	☐
Rectum	☐	☐	☐	☐	☐	☐	☐
Eye	☐	☐	☐	☐	☐	☐	☐
Lung	☐	☐	☐	☐	☐	☐	☐
Esophagus	☐	☐	☐	☐	☐	☐	☐
Skin	☐	☐	☐	☐	☐	☐	☐

Please provide any other indication at your province not listed above for each treatment type (HDR IC, HDR IT, HDR IC+IT, HDR Plesio, LDR IC, LDR IT, LDR Eye Plaque) in the textbox below.

7. Did your data have consistent definition and codes for each treatment type from beginning of 2011 to end of 2020?

- ◯ Yes
- ◯ No

8. Please add in the textbox below the codes that you have used for each treatment type.

	Code
HDR IC	
HDR IT	
HDR Plesio	
LDR IC	
LDR IT	
LDR Eye plaque	

9. <u>If the answer to question (7) is No</u>, please provide information on the change(s)?

10. What is the total number of treatments given for each patient?
